# The BRD4 inhibitor JQ1 suppresses tumor growth by reducing c-Myc expression in endometrial cancer

**DOI:** 10.1186/s12967-022-03545-x

**Published:** 2022-07-28

**Authors:** Yingxin Pang, Gaigai Bai, Jing Zhao, Xuan Wei, Rui Li, Jie Li, Shunxue Hu, Lu Peng, Peishu Liu, Hongluan Mao

**Affiliations:** 1 Department of Obstetrics and Gynecology, Qilu Hospital of Shandong University, No.107 Wenhua West Road, Jinan, 250012 Shandong China; 2 Key Laboratory of Gynecology Oncology of Shandong Province, Qilu Hospital of Shandong University, Jinan, Shandong China; 3 Shandong Engineering Laboratory for Urogynecology, Qilu Hospital of Shandong University, Jinan, Shandong China; 4 Department of Clinical Laboratory, Qilu Hospital of Shandong University, Jinan, Shandong China; 5 Department of Pathology, Qilu Hospital of Shandong University, Jinan, Shandong China

**Keywords:** Endometrial cancer, BRD4, JQ1, Cell apoptosis, Cell cycle arrest, c-Myc

## Abstract

**Background:**

Endometrial cancer (EC) is the most common gynecological malignancy in developed countries. Efficacy of the bromodomain 4 (BRD4) inhibitor JQ1 has been reported for the treatment of various human cancers, but its potential impact on EC remains unclear. We therefore aimed to elucidate the function of BRD4 and the effects of JQ1 in EC in vivo and in vitro.

**Methods:**

The mRNA expression of *BRD4* was evaluated using datasets from The Cancer Genome Atlas (TCGA) and Gene Expression Omnibus (GEO). BRD4 protein expression in EC tissues was measured using immunohistochemistry (IHC) assays. The effects of JQ1 on EC were determined by using MTT and colony formation assays, flow cytometry and xenograft mouse models. The underlying mechanism was also examined by western blot and small interfering RNA (siRNA) transfection.

**Results:**

BRD4 was overexpressed in EC tissues, and the level of BRD4 expression was strongly related to poor prognosis. The BRD4-specific inhibitor JQ1 suppressed cell proliferation and colony formation and triggered cell apoptosis, cell cycle arrest, and changes in the expression of proteins in related signaling pathways. Moreover, JQ1 decreased the protein expression of BRD4 and c-Myc, and knockdown of *BRD4* or *c-Myc* reduced the viability of EC cells. Intraperitoneal administration of JQ1 (50 mg/kg) significantly suppressed the tumorigenicity of EC cells in a xenograft mouse model.

**Conclusion:**

Our results demonstrate that BRD4 is a potential marker of EC and that the BRD4 inhibitor JQ1 is a promising chemotherapeutic agent for the treatment of EC.

**Supplementary Information:**

The online version contains supplementary material available at 10.1186/s12967-022-03545-x.

## Background

Endometrial cancer (EC) is the most common gynecological malignancy in developed countries. In 2021, approximately 66,570 new cases of EC and 12,940 deaths were expected in the United States [1]. Unlike other gynecological cancers, the incidence and mortality of EC continue to rise. The mainstay treatment for high-risk EC is surgery combined with chemotherapy and/or radiotherapy [2]. However, prognosis is still poor for advanced or metastatic disease, and effective treatment remains challenging. In 2018, an open-label international randomized phase 3 trial (PORTEC-3) including 660 patients showed that traditional chemoradiotherapy did not improve quality of life or prolong 5-year overall survival compared with pelvic radiotherapy alone for women with high-risk EC [3].

Comprehensive genomic approaches can identify genetic and molecular abnormalities to aid the prediction of prognosis and validation of new drug-targeting strategies. These technologies may finally enable precision medicine for individual patients by allowing the selection of specific treatments based on molecular parameters. One gene that is commonly dysregulated in malignant tumors is c-Myc. c-Myc encodes an oncogenic transcription factor that regulates the expression of approximately 30% of all human genes [4, 5], including genes involved in the cell cycle, proliferation and apoptosis [6, 7]. A recent study showed that c-Myc is overexpressed in > 70% of endometrial tumors [8]. However, finding direct c-Myc inhibitors is challenging [4].

Bromodomain 4 (BRD4) is a member of the bromodomain and extraterminal (BET) family and acts as an epigenetic reader by recruiting transcription complexes to specific sites of chromatin to initiate transcription of oncogenic drivers [9]. BRD4 facilitates the recruitment of positive transcription elongation factor (p-TEFb) to activate c-Myc transcription [10]. Aberrant BRD4 expression may contribute to the progression of multiple cancers, including hematological malignancies and several types of solid cancers [11]. Promising antitumor efficacy of BET inhibitors has been reported, and clinical trials are ongoing for a variety of cancers [12]. In particular, effects of BET inhibitors on uterine serous carcinoma, an aggressive type of type II EC, have been reported [13]. The best-studied BET inhibitor is JQ1, a potent and specific BRD4 inhibitor with antitumor and anti-inflammatory activities [14]. However, the protein expression profile of BRD4 in EC patients has not been characterized, and the mechanism by which BRD4 impacts EC progression remains unknown.

In the present study, we analyzed data from The Cancer Genome Atlas (TCGA) and Gene Expression Omnibus (GEO) as well as clinically isolated endometrial tissues and found that BRD4 is aberrantly expressed in EC tissue compared with normal endometrial tissue. In addition, overexpression of BRD4 was associated with relatively poor prognosis. To explore the effects of the BRD4 inhibitor JQ1 on tumor growth and the underlying mechanism, experiments were performed using EC cell lines and EC xenograft models. Our findings indicate that BRD4 is an oncogene and prognostic predictor in EC that promotes cell proliferation by regulating c-Myc. Accordingly, BRD4 inhibitors could be a promising therapeutic strategy for EC.

## Materials and methods

### Drugs and reagents

Dimethyl sulfoxide (DMSO) and 3-(4,5-dimethylthiazol-2-yl)-2,5-diphenyltetrazolium bromide (MTT) were purchased from Sigma-Aldrich (St. Louis, MO, USA). The BRD4 inhibitors, JQ1, I-BET151 and OTX015, were all purchased from MedChemExpress (Shanghai, China), dissolved in DMSO, and stored in small aliquots – 20 ℃.

### Cell lines and cell culture

Human EC cell lines (HEC-1A, Ishikawa, RL-95 and An3C cells) were purchased from the Cell Bank of the Chinese Academy of Sciences (Shanghai, China). HEC-1A cells were cultured in McCoy’s 5A medium (Thermo Fisher Scientific Inc., Waltham, MA, USA) supplemented with 10% fetal bovine serum (FBS) (Biological Industries, Kibbutz Beit-Haemek, Israel) and 1 mM sodium pyruvate (Thermo Fisher Scientific Inc.). Ishikawa and An3C cells were grown in RPMI 1640 medium (Thermo Fisher Scientific Inc.) with 10% FBS. RL-95 cells were maintained in DMEM/F12 medium (Thermo Fisher Scientific Inc.) with 10% FBS. All cell culture media were supplemented with 100 U/ml penicillin and 100 μg/ml streptomycin (Thermo Fisher Scientific Inc.). Cells were cultured at 37 ℃ in a 5% CO_2_ atmosphere. For in vitro experiments, BET inhibitors were added to cultured cancer cells at indicated concentrations. Equivalent concentrations of DMSO were added to cells as controls.

### Patients and specimens

Fresh endometrial tissue samples from 50 patients with EC (Type I, n = 33; Type II, n = 17) and 14 patients with leiomyoma who had undergone hysterectomy between January 2010 and June 2012 were obtained from the Department of Gynecology and Obstetrics of Qilu Hospital. Staging and histological subtyping were performed according to the 2009 guidelines of the International Federation of Gynecology and Obstetrics. Fully informed written consent was acquired from the patients before the collection of tissue samples. This study was approved by the Ethics Committee of Qilu Hospital of Shandong University (KYLL-2019(KS)-376). The clinical characteristics of the EC patients are listed in Table 1. All patients were followed up until June 2019. The patients in this study had no history of other previous cancers and did not undergo any preoperative chemotherapy, radiotherapy, or other hormonal therapies. Overall survival (OS) was defined as the time between the date of surgery and the date of patient death directly due to EC or last follow-up. Some tissues were preserved immediately in liquid nitrogen for subsequent western blot assays. All samples were fixed in 4% paraformaldehyde for 24 h, dehydrated, embedded in paraffin blocks, and sectioned (4 μm thickness) for immunohistochemistry (IHC) assays.

**Table 1:** Characteristics of EC patients

Variables	Values
Age, median (range), years	54 (43–67)
Surgery, *n* (%)	
TH/BSO alone	22 (44%)
TH/BSO plus lymphadenectomy	28 (56%)
FIGO grade, *n* (%)	
Grade 1	27 (54%)
Grade 2	14 (28%)
Grade 3	9 (18%)
Pathologic type, *n* (%)	
Type I	33(66%)
Type II	17(34%)
Stage, *n* (%)	
I	29(58%)
II	14(28%)
III	7(14%)
Lymphovascular space involvement, *n* (%)	11(22%)
Deep (≥ 50%) myometrial invasion, *n* (%)	15(30%)
Lymph node involvement, *n* (%)	9(18%)
Adjuvant therapy, *n* (%)	22(44%)
Chemotherapy alone	10(20%)
Chemotherapy + Brachytherapy	12(24%)
Median follow up time, (95% CI), months	65(15–79)
Recurrence, *n* (%)	8(16%)

### Immunohistochemistry assays

Endometrial tissue sections were deparaffinized with xylene and rehydrated in different concentrations of ethanol. Hydrogen peroxide was used to block endogenous peroxidase activity, and nonspecific antigens were blocked by incubation in goat serum for 30 min. The sections were then incubated with primary antibodies overnight at 4 °C. The primary antibody against BRD4 was purchased from Abcam (1:50 dilution, Cambridge, UK). After sequential incubation with biotin-labeled goat anti-rabbit IgG polymer and horseradish peroxidase-labeled streptavidin for 30 min each, positive signals were detected using 3,3′-diaminobenzidine (DAB) substrate (Zhongshan Jinqiao Biotechnology, Beijing, China) following the manufacturer’s recommendations. Finally, the stained tissues were evaluated and scored by two blinded investigators.

### Scoring of immunoreactivity

Strong positive staining was defined as a brown signal in the nucleus or cytoplasm; moderate staining as a yellow–brown reaction; and weak staining as a light-yellow reaction. The staining intensity was graded as follows: 0 = no staining; 1 = weak staining; 2 = moderate staining; and 3 = strong staining. The percentage of tumor cell staining was graded based on the following criteria: 0 = no staining; 1 =  ≤ 10% positive tumor cells; 2 = 11–50% positive tumor cells; 3 = 51–80% positive tumor cells; and 4 =  ≥ 81% positive tumor cells. The staining score was calculated by multiplying the intensity score by the quantity score and ranged from 0 to 12. Scores ≥ 8 indicated overexpression of BRD4 in endometrial tissues.

### Proliferation assays

Cells (2000 cells in 100 μl/well) were propagated in 96-well culture plates overnight and then exposed to the indicated concentrations of JQ1, OTX015, I-BET151 or transfected with siRNA or BRD4 overexpression lentivirus. After treatment for indicated time, 10 μl of MTT solution (5 mg/ml) was added to each well, and the plates were incubated at 37 ℃ for another 4 h. Then, the medium was removed, and DMSO (100 μl/well) was added to dissolve the formazan product. The absorbance of each well was measured at 570 nm using a microplate reader (Tecan Group Ltd., Männedorf, Switzerland). Three replicate wells were included for each experiment, and the experiments were performed in triplicate.

### Colony formation assay

Cells (800 cells in 2 ml/well) were seeded in 6-well plates and cultured for 48 h. Next, the cells were treated with JQ1 or OTX015 at the indicated concentrations for 14 days at 37 ℃ in 5% CO_2_. The cells were fixed with paraformaldehyde for 20 min and stained with crystal violet (Beyotime, Beijing, China) for 30 min, and colonies (> 50 cells) were counted.

### Protein extraction and western blot

RIPA buffer with protease inhibitor cocktail, PMSF (0.1 mM) and NaF (10 mM) was used to lyse cells treated with JQ1 or transfected with siRNA. Protein concentrations were determined by the BCA protein assay kit (Beyotime, Beijing, China). Equal amounts of protein (30 μg) were separated by SDS-PAGE on a 10% gel and transferred to a nitrocellulose membrane. After blocking with 5% defatted milk for 1 h at room temperature, the membrane was incubated overnight at 4 ℃ with primary antibodies against BRD4 (Abcam), c-Myc, CDK4, Bax (Santa Cruz, CA, USA), β-Actin, cleaved-Caspase-3 (cl-Caspase3), PARP, cleaved-PARP (cl-PARP), CDC25A, CyclinD1, P21, Rb, phosphorylated Rb (p-Rb), PI3K, AKT, phosphorylated AKT (p-AKT), mTOR, phosphorylated mTOR (p-mTOR) (Cell Signaling Technology, MA, USA). Secondary antibodies were purchased from Cell Signaling. Protein bands were detected using an enhanced chemiluminescent substrate (Thermo Fisher Scientific Inc.). ImageJ software (National Institutes of Health, USA) was used to quantify the immunoblots. β-Actin served as a control.

### Apoptosis assay

Apoptosis of JQ1 or OTX015-treated EC cells or cells infected with lentivirus was detected by flow cytometry. Cells were washed with PBS, digested with trypsin without EDTA and resuspended in 100 μl of binding buffer. Next, the cells were stained with 5 μl of Annexin V-FITC and 5 μl of propidium iodide (PI) (BD, NJ, USA). After incubation at room temperature in the dark for 15 min, another 400 μl of binding buffer was added to each tube. Cell apoptosis was analyzed by flow cytometry (BD Biosciences, San Jose, CA, USA) according to the manufacturer’s instructions.

### Cell cycle assay

Cell cycle analysis was performed by flow cytometry. Synchronized cells treated with JQ1 or OTX015 for 24 h were washed with PBS, digested with trypsin with EDTA, and fixed in 75% cold ethanol overnight at 4 ℃. The cells were washed twice with PBS and stained with 400 μl of PI/RNase Staining Buffer (BD Biosciences) at room temperature in the dark for 30 min. The cell cycle was then analyzed by flow cytometry according to the manufacturer’s instructions.

### Transient transfection of siRNA

Small interfering RNAs (siRNAs) targeting human *BRD4* or *c-Myc* and silencer negative control siRNAs were constructed by GenePharma (Shanghai, China). Cells were seeded into 6-well plates at 2.5 × 10^5^ cells/well and incubated overnight before transfection with 150 nM (final concentration) *BRD4* or *c-Myc* siRNA using Lipofectamine 3000 (Invitrogen) according to the manufacturer’s instructions. For western blot assays, the cells were collected 72 h following transfection. For MTT assays, cells were collected on days 1–5 following transfection.

### Exogenous BRD4 overexpression

To establish stable cell lines, lentivirus was purchased from GeneChem (Shanghai, China). Ishikawa cells were infected with the pUbi-BRD4 and pUbi-NC lentivirus. After 48 h, cells were screened with 2 μg/mL puromycin (Solarbio, Beijing, China) for 1–2 weeks to obtain stable cell lines.

### Tumor xenograft experiments

Animal experiments were approved by Institutional Animal Care and Use Committees of Qilu Hospital of Shandong University. Ishikawa cells (1 × 10^7^ cells in 100 μl of PBS) were subcutaneously injected into the left armpits of 5-week-old nude mice. When the tumor volume reached approximately 50 mm^3^, the mice were randomly allocated to the treatment or control group. Depending on the group, JQ1 (50 mg/kg/d) or placebo was administered intraperitoneally daily for 3 weeks. Body weight and tumor volume were measured every other day. Tumor volume was calculated using the following formula: (length × width^2^)/2. Blood was sampled by extirpation of the eyeball before death and used for routine blood examination. The mice were sacrificed 21 days after initiating JQ1 treatment, and tumors were immediately removed and weighed.

### Statistical analyses

Survival probabilities were calculated by the Kaplan–Meier method. Because the number of events was low for overall survival, we could not evaluate the independent prognostic value of BRD4 in a multivariable model adjusting for BMI, disease stage, histological type, and histological grade simultaneously. All experiments were repeated at least three times. Student’s *t*-test was used to compare differences between two groups. The results are presented as the means ± standard deviations (SD). All statistical tests were two-sided; *P < 0.05 was considered statistically significant, **P < 0.01 moderately significant, and ***P < 0.001 highly significant. GraphPad Prism Version 7.00 was used to perform the statistical analyses.

## Results

### BRD4 is upregulated in EC tissues, and elevated BRD4 predicts poor prognosis

**Fig. 1:**
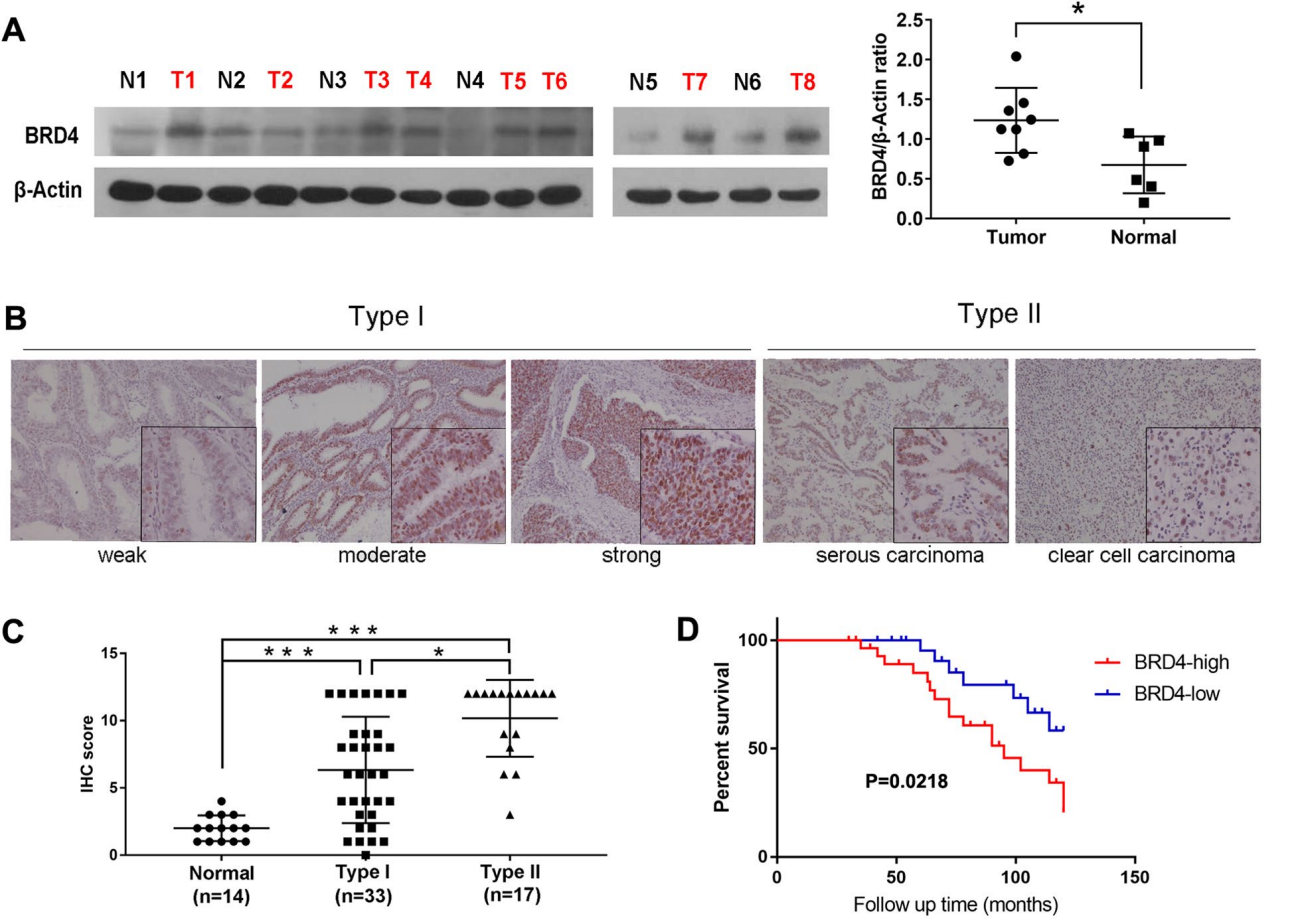
The expression of BRD4 was upregulated in EC tissues and was negative related with patient prognosis. **A** The expression levels of BRD4 measured by western blot analysis in frozen EC tissues (n = 8) and normal endometrial tissues (n = 6). β-Actin served as the loading control. **B** Representative IHC staining of BRD4 in EC tissues. **C** The expression levels of BRD4 in normal endometrial tissues (n = 14), type I (n = 33) and type II (n = 17) EC tissues. **D** Kaplan–Meier survival analysis of BRD4 expression in EC patients

Tissue samples derived from 50 patients with EC and 14 patients with leiomyoma undergoing hysterectomy between January 2010 and June 2012 were used to validate and estimate the potential role of BRD4. To determine the expression levels of BRD4 in EC tissues and normal endometrial samples, western blot and IHC analyses were performed. As expected, BRD4 protein expression was upregulated in EC tissue (n = 8) compared with normal endometrial tissue (n = 6) (Fig. 1A). IHC staining demonstrated that BRD4 protein expression was primarily located in the nucleus (Fig. 1B). In addition, high BRD4 expression was significantly associated with serous carcinoma and clear cell carcinoma (Fig. 1C). The stained EC tissue samples were scored and divided into groups with high (n = 28) or low (n = 22) expression of BRD4. Strong nucleic BRD4 expression was significantly associated with shorter EC patient survival in Kaplan–Meier analysis (*P* = 0.0218; Fig. 1D).

**Fig. 2:**
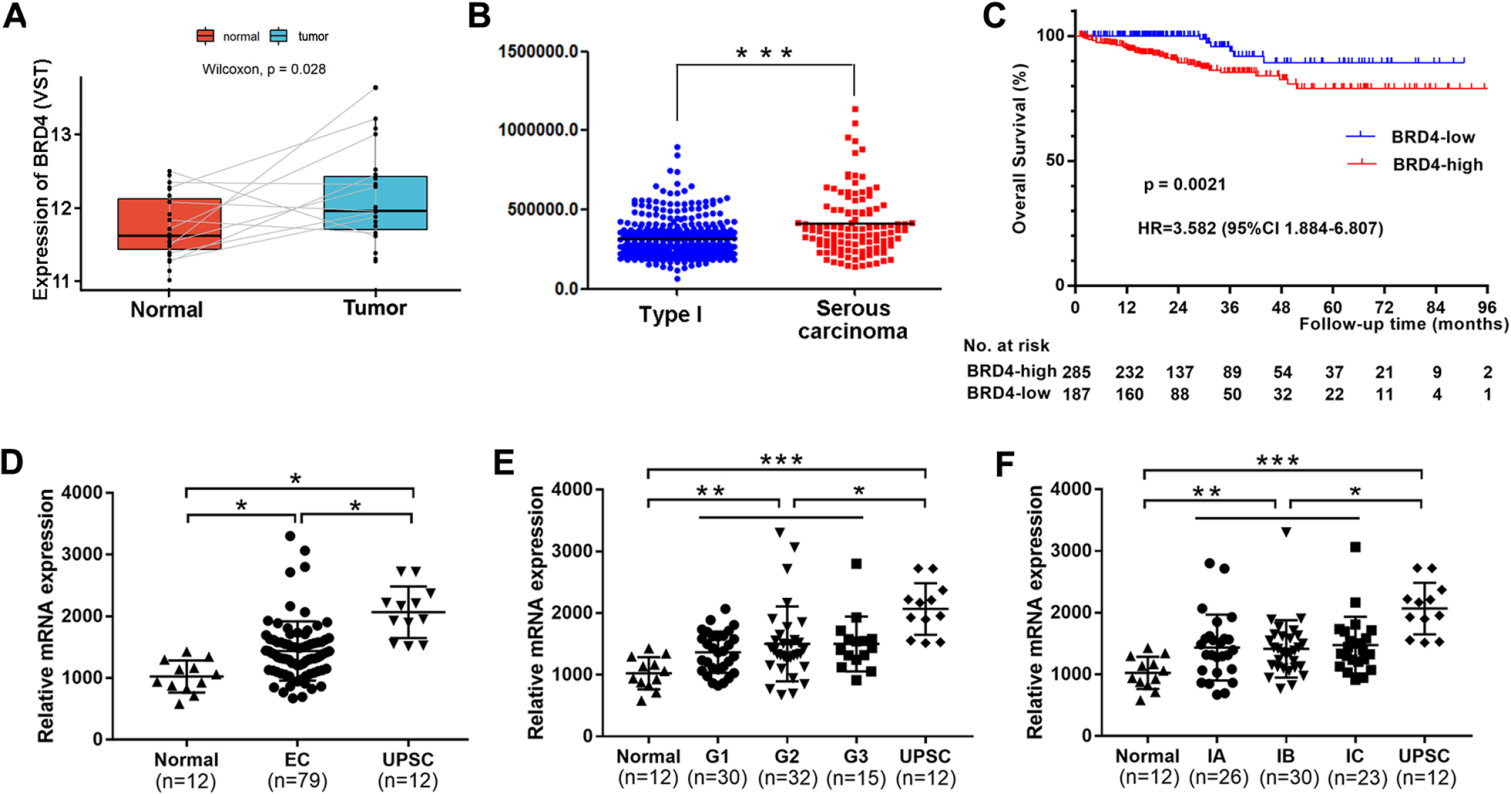
The expression of BRD4 and Kaplan–Meier survival analysis in EC patients were evaluated by the TCGA and GEO database. **A** The expression of BRD4 in paired samples (EC tissues and its adjacent normal tissues) by the TCGA database. **B** The expression of BRD4 in Type I and Type II EC patients by the TCGA database. **C** Kaplan–Meier survival analysis of BRD4 expression in EC patients by the TCGA database. **D** The expression of BRD4 in normal endometrial tissues, endometrioid EC tissues and uterine papillary serous carcinoma by the GEO database. **E** The expression of BRD4 in different grades of EC by the GEO database. **F** The expression of BRD4 in different FIGO stages of EC by the GEO database

TCGA and the GSE17025 dataset were used to further evaluate the expression of BRD4 and its prognostic value in EC. As shown in Fig. 2A, a paired-comparison test of data from TCGA showed that BRD4 expression was higher in EC cancer tissues than in corresponding adjacent tissues. In addition, BRD4 expression was significantly higher in serous carcinoma, a form of type II EC, than in type I EC (Fig. 2B). The cohort from TCGA was then divided into groups with high or low BRD4 expression. Compared with the low BRD4 expression group, OS was significantly lower in the high BRD4 expression group (*P* = 0.0021, Fig. 2C). Similar trends were observed in the analyses of the GSE 17025 dataset, as shown in Fig. 2D–F. However, in the Type I EC group, there were no significant differences in BRD4 expression according to grade or stage in TCGA and the GSE 17025 dataset. These results suggest that elevated BRD4 expression is a negative prognostic factor for EC patients.

### BET inhibitors reduce the proliferation of EC cells

**Fig. 3:**
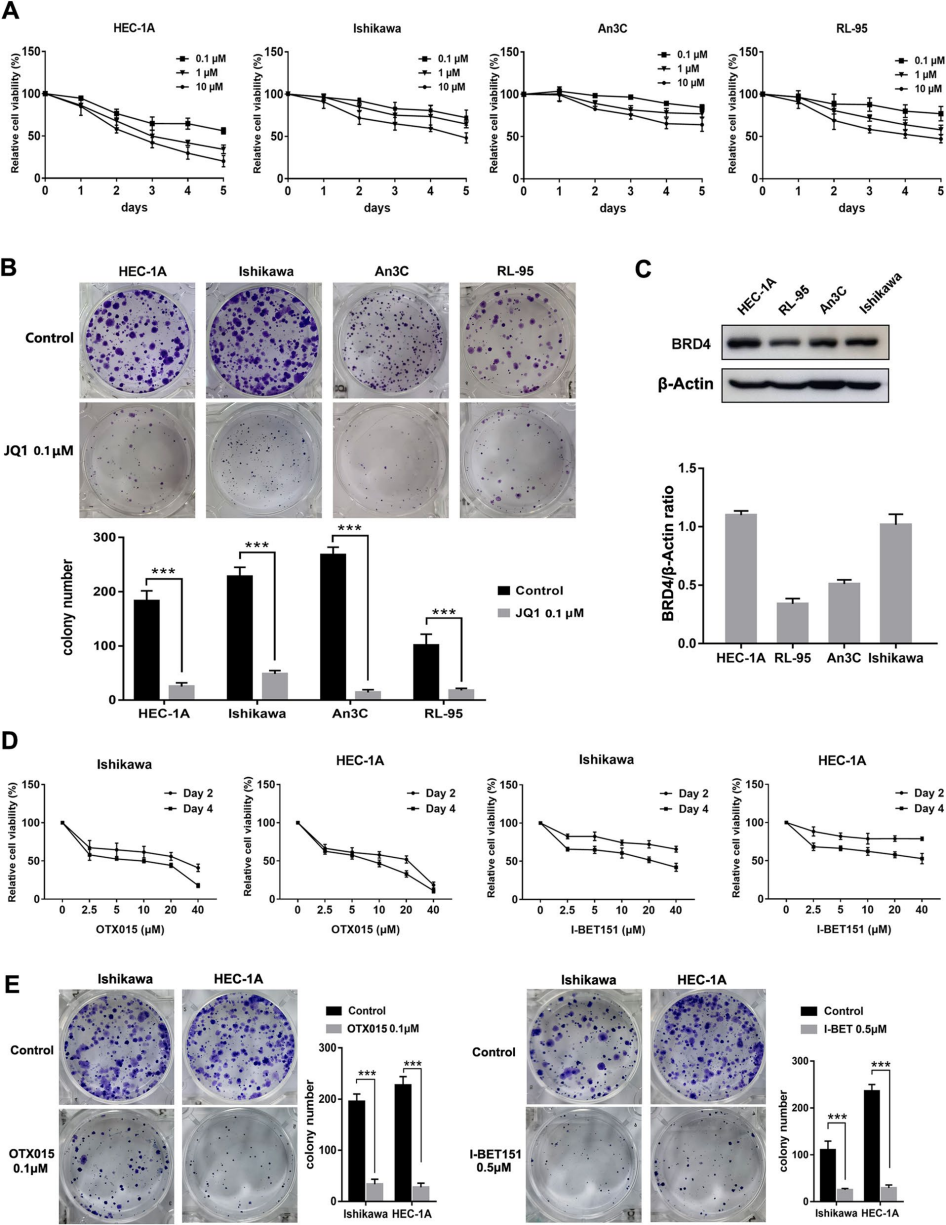
BET inhibitors treatment suppressed EC cell growth. **A** EC cells were treated with 0–10 µM JQ1 for 1–5 days. The cell vaibility of JQ1 was measured using MTT assay. **B** EC cells were treated with DMSO or 0.1 µM JQ1 for two weeks. The growth inhibition effects of JQ1 were measured using cell colony formation assay in four EC cell lines. **C** The relative expression levels of BRD4 were measured by western blot analysis in four EC cell lines. β-Actin served as the loading control. **D** EC cells were treated with 0–40 µM OTX015 or I-BET151 for 2 or 4 days. The growth inhibition was measured using MTT assay. **E** EC cells were treated with DMSO or 0.1 µM OTX015 (left side) or 0.5 µM I-BET151 (right side) for two weeks. The growth inhibition effects were measured using cell colony formation assay in Ishikawa and HEC-1A cell lines. Data shown are mean ± SD from three independent experiments

MTT and colony formation assays were used to detect the inhibitory effect of JQ1 on the proliferation of EC cells. The MTT assays revealed that JQ1 inhibited cell growth in a dose-dependent and time-dependent manner in all four EC cell lines (Fig. 3A). JQ1 concentration of 1.0 µM was sufficient to completely inhibit the growth of the two most sensitive cell lines, HEC-1A and Ishikawa, which were therefore chosen for subsequent experiments. Colony formation assays showed that treatment with JQ1 for 14 days at a dose as low as 0.1 µM JQ1 inhibited colony formation by EC cells (Fig. 3B). Low doses of JQ1 (0.1 µM) reduced colony numbers, while 1.0 µM JQ1 led to almost complete inhibition of colony formation (data not shown). Detection of the expression of BRD4 in the four EC cell lines by western blot showed that BRD4 was highly expressed in HEC-1A and Ishikawa cell lines, which were relatively sensitive to JQ1 (Fig. 3C). To verify the role of BRD4 in EC cells, we performed experiments using two other common BET inhibitors, I-BET151 and OTX015. MTT assay demonstrated that OTX015 has shown a significant anti-proliferative activity against EC cells, superior to that of I-BET151 (Fig. 3D). Combined with literature reports, OTX015 has been used in several clinical trials. Therefore, we used OTX015 for the subsequent experiments. Similarly, OTX015 and I-BET151 suppressed cell colony formation in EC cells (Fig. 3E). Taken together, our results demonstrate that BET inhibitors reduce the proliferation of human EC cells and that the sensitivity to JQ1 depends on the expression level of BRD4.

### BET inhibitors induce apoptosis of EC cells

**Fig. 4:**
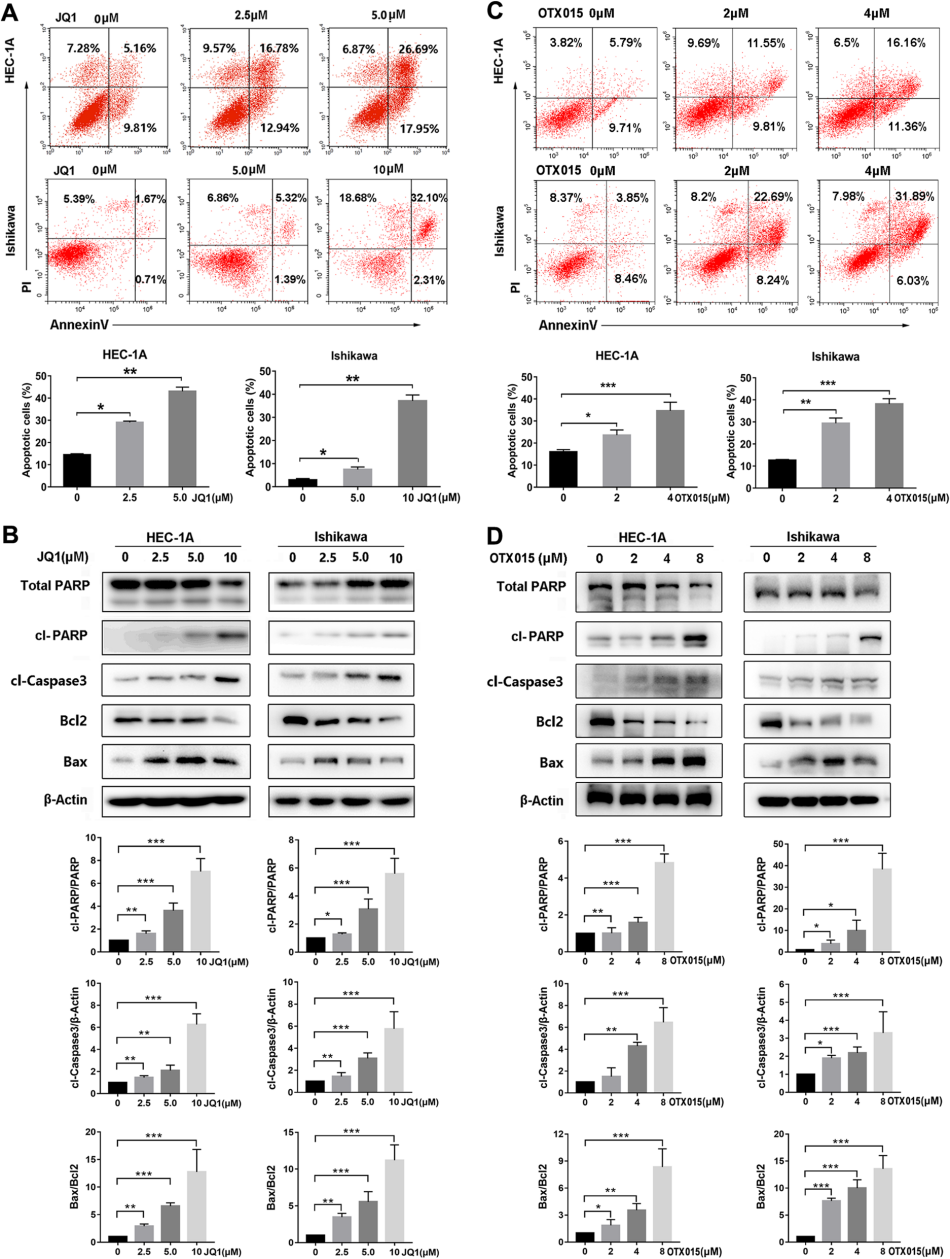
BET inhibitors induced apoptosis in EC cells. **A** HEC-1A and Ishikawa cells were treated with DMSO, 2.5 µM, 5 µM or 10μM JQ1 for 48 h. Cells were stained with Annexin V/FITC and PI, and then cell apoptosis was measured by flow cytometry. Data are shown in the histogram. Data shown are mean ± SD from three independent experiments. **B** HEC-1A and Ishikawa cells were treated with DMSO, 2.5 µM, 5 µM or 10μM JQ1 for 48 h. Protein expressions of total PARP, cleaved PARP, cleaved Caspase3, Bcl-2, Bax, and β-Actin were determined by western blot assay. β-Actin served as the loading control. The protein bands were quantified using ImageJ software and standardized by the β-Actin protein level. Data shown are mean ± SD from three independent experiments. **C** HEC-1A and Ishikawa cells were treated with indicated concentrations of OTX015 for 48 h. Cell apoptotic rates were measured by flow cytometry. **D** HEC-1A and Ishikawa cells were treated with indicated concentrations of OTX015 for 48 h. Protein expressions of total PARP, cleaved PARP, cleaved Caspase3, Bcl-2, Bax, and β-Actin were determined by western blot assay

To investigate the mechanism underlying the antiproliferative effects of BET inhibitors in EC cells, we analyzed cell apoptosis and apoptotic-signaling pathways using flow cytometry and western blot. Compared with the control group, JQ1 treatment for 48 h increased early and late apoptosis of EC cells (Fig. 4A). For HEC-1A and Ishikawa cells, the apoptosis rates were 43% and 11% in the groups treated with 5 µM JQ1, respectively, compared with 14% and 5% in the corresponding control groups. Consistent with the flow cytometry analysis, JQ1 activated caspase-3 and induced PARP cleavage (Fig. 4B). JQ1 also led to upregulation of the proapoptotic protein Bax and downregulation of the antiapoptotic protein Bcl-2 in EC cells. Like JQ1, OTX015 regulated expression of Bcl-2/Bax, and triggered cell apoptosis (Fig. 4C and D).

### JQ1 blocks the PI3K/AKT/mTOR pathway

**Fig. 5:**
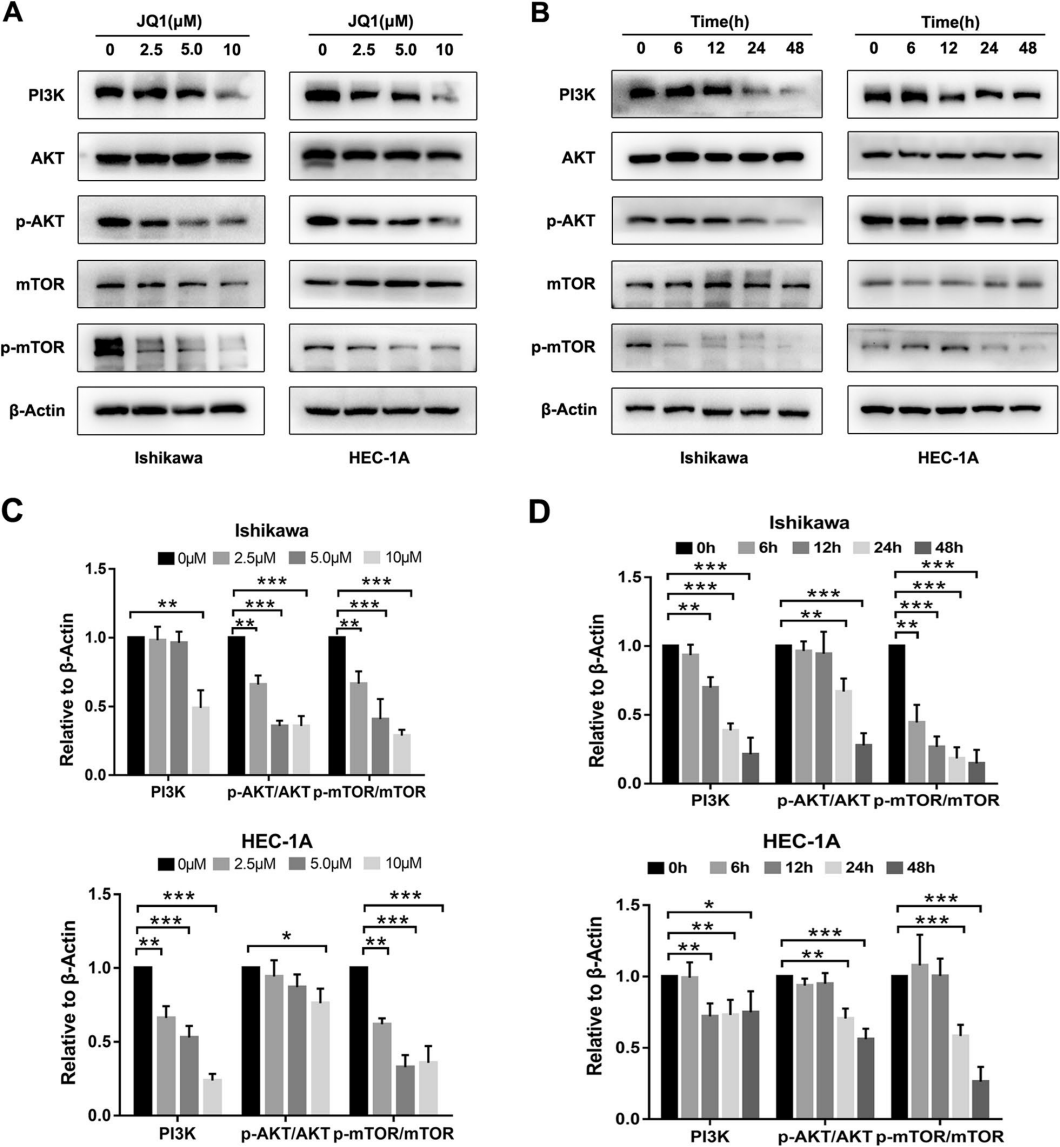
JQ1 blocked PI3K/AKT/mTOR pathway in EC cells. **A** HEC-1A and Ishikawa cells were treated with DMSO, 2.5 µM, 5 µM and 10 µM JQ1 for 48 h. **B** HEC-1A and Ishikawa cells were treated with 10 µM JQ1 for indicated times. Protein expressions of PI3K, AKT, phosphorylated AKT, mTOR, phosphorylated mTOR and β-Actin were determined by western blot assay. β-Actin served as the loading control. **C** and **D** The relative expression levels of indicated proteins were measured by western blot analysis. β-Actin served as the control

Given the important role of the PI3K/AKT/mTOR signaling pathway in cell growth and proliferation, we further investigated the impact of JQ1 on this pathway by western blot. As shown in Fig. 5A-D, in both HEC-1A and Ishikawa cells, JQ1 downregulated the total expression of PI3K but had no effect on the total expression of AKT or mTOR. However, JQ1 significantly downregulated the phosphorylation of AKT and TOR in a time- and dose-dependent manner.

### BET inhibitors suppress the cell cycle of EC cells in G1 phase

**Fig. 6:**
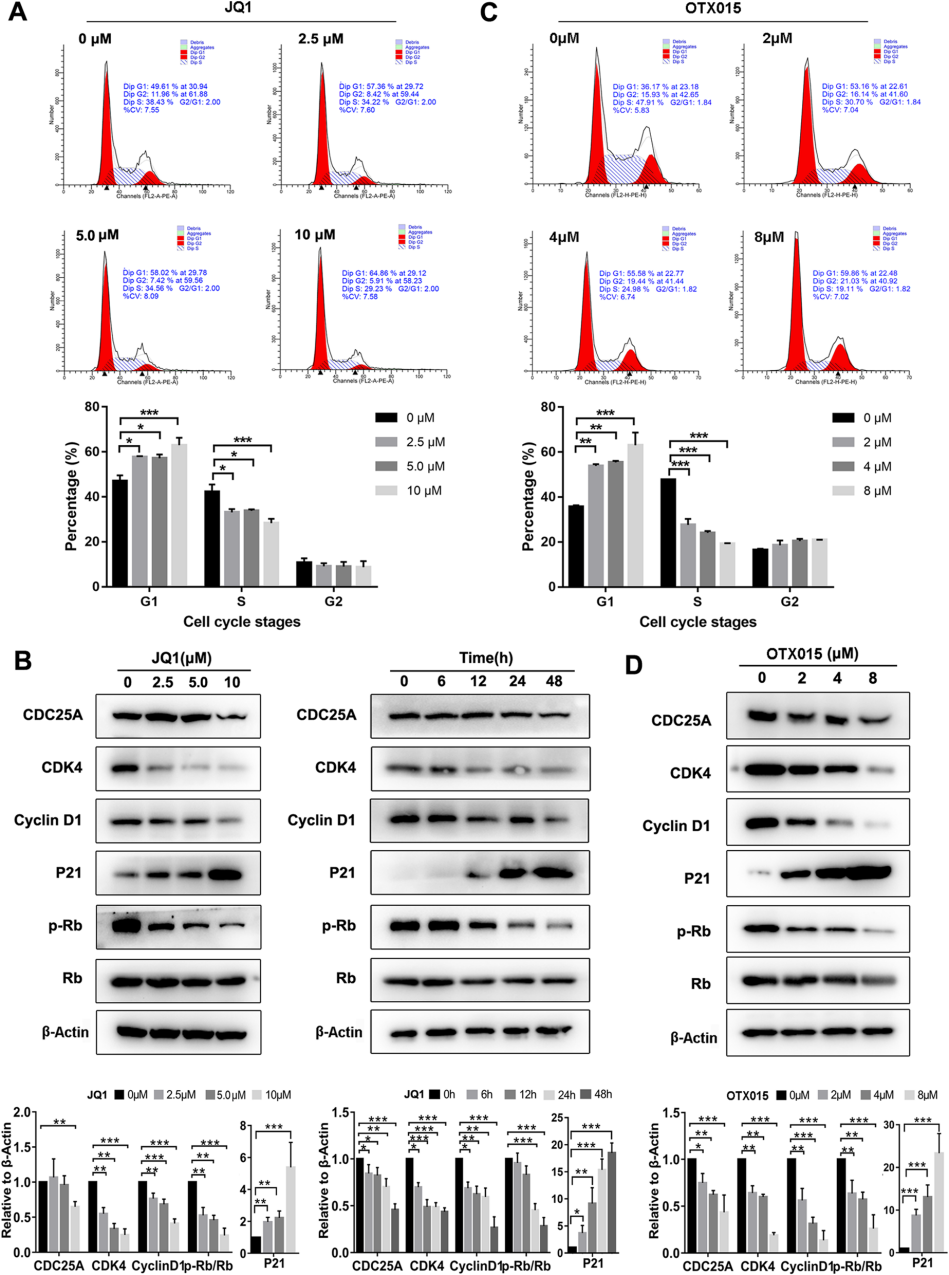
BET inhibitors suppressed the cell cycle of EC cells in G1 phase in ishikawa cells. **A** Ishikawa cells were treated with DMSO, 2.5 µM, 5 µM and 10 µM JQ1 for 24 h. Cells were stained with PI and analyzed by flow cytometry. Data are shown in the histogram. Data shown are mean ± SD from three independent experiments. **B** Ishikawa cells were treated with JQ1 (0, 2.5, 5 and 10 µM) for 24 h (left) or 10 µM JQ1 for indicated times (right). Protein expressions of CDC25A, CDK4, CyclinD1, P21, phosphorylated Rb, Rb and β-Actin were determined by western blot assay. β-Actin served as the loading control. **C** Ishikawa cells were treated with OTX015( 0, 2, 4 and 8 µM) for 24 h. Cells were stained with PI and analyzed by flow cytometry. **D** Ishikawa cells were treated with indicated concentrations of OTX015 for 24 h. Protein expressions of CDC25A, CDK4, CyclinD1, P21, phosphorylated Rb, Rb and β-Actin were determined by western blot assay. β-Actin served as the loading control

The effect of JQ1 and OTX015 on the cell cycle in EC cells was evaluated by flow cytometry and western blot. Flow cytometry showed that both of JQ1 and OTX015 suppressed the cell cycle in G1 phase in Ishikawa cells (Fig. 6A and C) but not HEC-1A cells (Additional file 1: Fig. S1). Treatment with different concentrations of JQ1 increased the share of Ishikawa cells in G1 phase from 47.03 ± 2.06 to 62.96 ± 2.70%. As for OTX015, the rate of G1 phase in the control group was 35.32 ± 3.14%, whereas, the rate rose to 58.45 ± 6.83% in OTX015 group of high concentrations. To evaluate the underlying mechanism, we analyzed cell cycle signaling pathways by western blot. JQ1 suppressed the expression of CDC25A, CDK4, and CyclinD1 and phosphorylation of Rb. By contrast, JQ1 upregulated P21 expression (Fig. 6B and D).

### Down-regulation of BRD4 influences c-Myc expression and PI3K/AKT/mTOR pathway

**Fig. 7:**
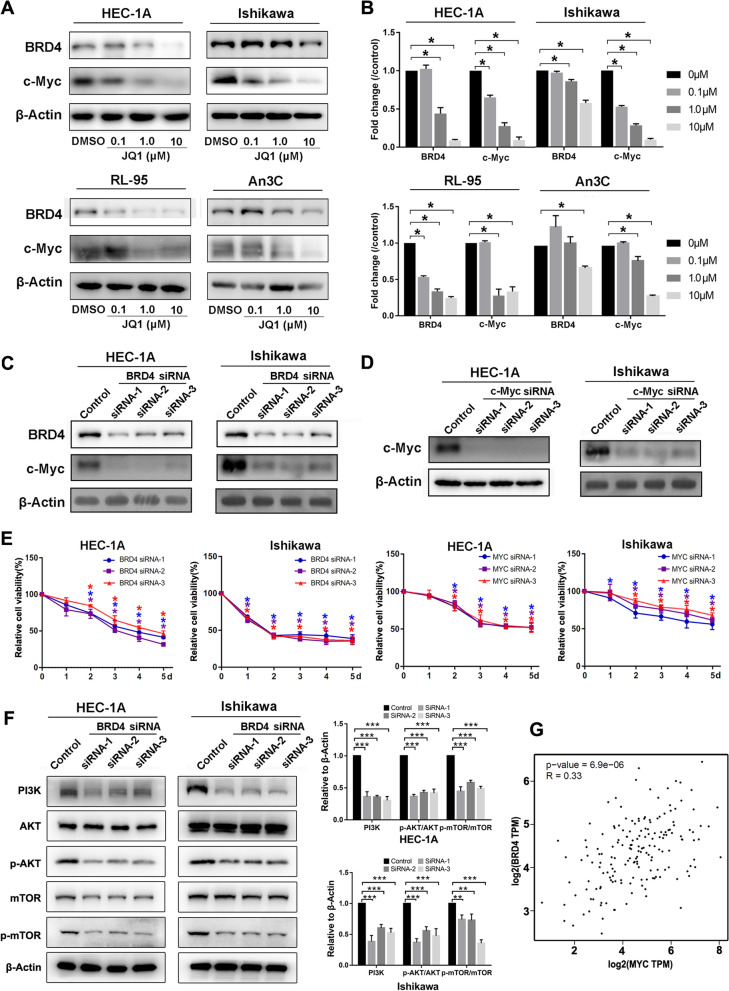
JQ1 and BRD4 knockdown suppressed expression of c-Myc and PI3K/AKT/mTOR pathway. **A** HEC-1A, Ishikawa, RL-95 and An3C cells were treated with DMSO, 0.1 µM, 1 µM and 10 µM JQ1 for 48 h. Protein expressions of BRD4, c-Myc and β-Actin were determined by western blot assay. β-Actin served as the loading control. **B** The protein bands were quantified using ImageJ software and standardized by the β-Actin protein level. Data shown are mean ± SD from three independent experiments. **C** HEC-1A and Ishikawa cells were transfected with 150 nM *BRD4* siRNA using Lipofectamine 3000. After 72 h, cells were collected for dermining protein expression of BRD4 and c-Myc using western blot assay. β-Actin served as the loading control. **D** Cells were transfected with *c-Myc* siRNA. After 72 h, cells were collected for dermining protein expression of c-Myc using western blot assay. **E** HEC-1A and Ishikawa cells were transfected with *BRD4* or *c-Myc* siRNA. Cell proliferation rate was determined by MTT assay on days 1–5 following transfection. **F** HEC-1A and Ishikawa cells were transfected with *BRD4* siRNA for 72 h. Protein expressions of PI3K, AKT, phosphorylated AKT, mTOR, phosphorylated mTOR and β-Actin were determined by western blot assay. β-Actin served as the loading control. **G** Pearson correlation between *BRD4* and *c-Myc* genes was performed using cBioPortal and Gene Expression Profiling and Interactive Analysis (GEPIA)

JQ1 has been reported to exert its antitumor effects by suppressing c-Myc expression in other solid tumors. We therefore examined the influence of JQ1 on BRD4 and c-Myc expression in EC cell lines. Western blot showed that JQ1 significantly reduced BRD4 and c-Myc protein expression (Fig. 7A and B). siRNA knockdown of *BRD4* also inhibited c-Myc transcription and cell proliferation, suggesting that JQ1 reduces c-Myc expression through inhibition of BRD4 (Fig. 7C). siRNA knockdown of* c-Myc* in EC cell lines inhibited cell growth (Fig. 7D and E), but the inhibitory effect was weaker than that elicited by treatment with 2.5 µM JQ1, suggesting that JQ1 may have effects other than repression of c-Myc expression. Western blot assay showed that knockdown of *BRD4* inhibited the PI3K/AKT/mTOR pathway, which was basically the same as JQ1 (Fig. 7F). Analysis of TCGA data using the online tool GEPIA (Gene Expression Profiling Interactive Analysis) showed that BRD4 expression is positively correlated with c-Myc expression in EC tissues (Fig. 7G).

### Overexpression of BRD4 partially reverses the effect of JQ1

**Fig. 8:**
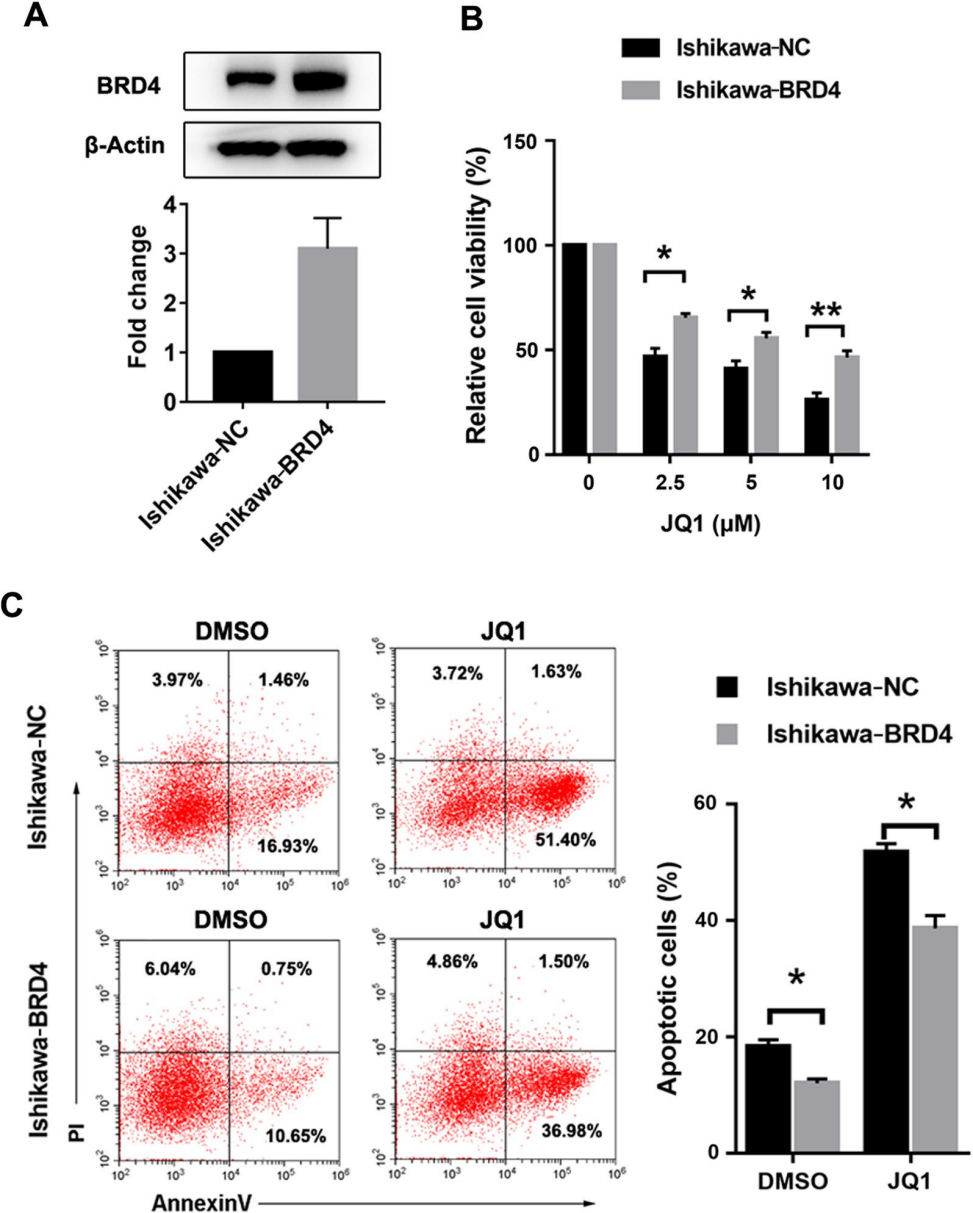
Overexpression of BRD4 reversed the effect of JQ1. **A** Protein expressions of BRD4 and β-Actin in Ishikawa-NC and Ishikawa-BRD4 overexpression cells were determined by western blot assay. β-Actin served as the loading control. **B** Ishikawa-NC and Ishikawa-BRD4 overexpression cells were treated with 0–10 µM JQ1 for 48 h. The cell viability of JQ1 was measured using MTT assay. **C** Cells were stained with Annexin V/FITC and PI, and then cell apoptosis was measured by flow cytometry. Data are shown in the histogram. Data shown are mean ± SD from three independent experiments

We established Ishikawa-NC cells as well as Ishikawa-BRD4 overexpression cells. BRD4 protein level was significantly increased in stable cells with the overexpression vector (Fig. 8A). Compared with control cells, the cell viability rate was higher in Ishikawa-BRD4 overexpression cells following the treatment with different concentrations of JQ1 (Fig. 8B). Cell apoptotic rates mediated by JQ1 were partially reversed by forced overexpression of BRD4 (Fig. 8C). Our data demonstrated that the antitumor effect of JQ1 to some extent was BRD4-dependent in endometrial cancer cells.

### JQ1 inhibits tumor growth in vivo in mouse xenograft models of EC

**Fig. 9:**
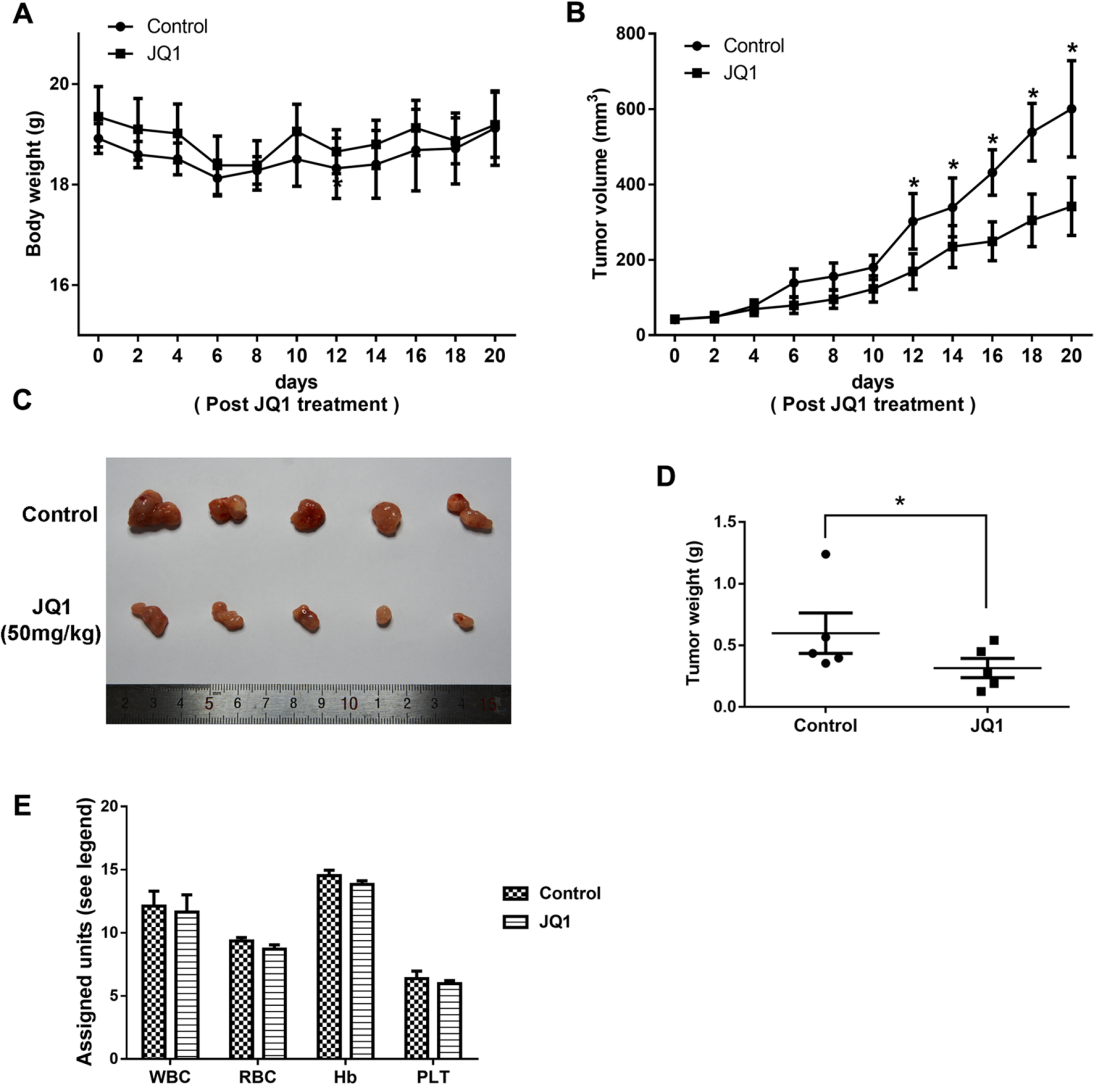
JQ1 suppressed tumor growth in vivo in mouse xenograft models of endometrial cancer. Body weight (**A**) and tumor volume (**B**) from Ishikawa xenografts were measured every 2 days after treatment with JQ1. **C** Representative photographs of tumors dissected from xenografts after treatment with JQ1 for 3 weeks. **D** Tumor weight was determined at time of sacrifice in placebo and JQ1-treated groups. **E** Comparison of white blood cells (WBC) (10^9^/L), erythrocytes (RBC) (10^12^/L), hemoglobin (Hb) (g/L) and platelet (PLT) (10^9^/L) levels in control and JQ1-treated mice. Data are represented as mean ± SEM (n = 5)

To further validate the suppressive effect of JQ1 in vivo, we used Ishikawa cells to construct mouse xenograft models. The xenograft models were then administered JQ1 (50 mg/kg/d) or placebo intraperitoneally daily for 3 weeks. There was no obvious difference in body weight between the two groups (Fig. 9A). JQ1 administration clearly reduced EC tumor growth in  vivo. Compared with the placebo group, tumor volume and tumor weight were smaller in the JQ1-treated group (Fig. 9B and D). The difference in tumor volume between the JQ1-treated group and the placebo group became significant on day 12. Representative EC tumors are shown in Fig. 9C. At the end of the experiment, the mean tumor weight was 598.8 ± 328.4 mg in the placebo group (n = 5) but 316.0 ± 156.3 mg in the JQ1-treated group (n = 5) (*P* < 0.05). Moreover, routine blood counts showed that JQ1 had no obvious toxicity with respect to hematopoietic function (Fig. 9E). Taken together, these data suggest that JQ1 suppresses EC progression in vivo.

## Discussion

There is a clear need for novel prognostic biomarkers for EC to ensure adequate risk stratification and to facilitate the development of individualized therapies. The goals of this study were threefold. The first and primary goal was to evaluate the expression of BRD4 in EC tissues and its implications for prognosis. Second, the antitumor effects of the BRD1 inhibitor JQ1 on EC were evaluated in vitro and in vivo. The third goal was to explore the potential mechanism(s) underlying the effects of JQ1 on endometrial tumors.

BRD4 is an epigenetic reader that has been implicated in the regulation of the development and progression of various cancers [11]. No published studies have evaluated the expression of BRD4 in clinically derived EC tissues or analyzed BRD4 as a prognostic factor. Overexpression of BRD4 has been reported in a variety of malignant tumor types, including breast cancer, hematological malignancies, and lung cancer [9, 15]. In the present study, western blot and IHC analyses of BRD4 protein expression in EC and normal endometrial tissues showed that BRD4 is overexpressed in endometrial tumors and that nuclear BRD4 expression levels are negatively correlated with overall patient survival time. These data indicate that BRD4 may be a useful adjunct tissue biomarker for postoperative risk stratification of EC patients. BRD4 expression was also a significant independent risk factor in multivariate analysis. The small number of patients is a limitation of this study. Because this was a single-center study with a limited sample size, univariate and multivariate analyses were not performed. However, our results are supported by analyses of BRD4 expression and its relationship with prognosis using TCGA and GEO datasets.

Mechanistically, JQ1 potently and specifically inhibits the binding of the BRD4 protein to chromatin in a competitive manner [16]. As a result, downstream genes of BRD4 cannot be transcribed [10]. Efficacy of JQ1 alone or in combination with other chemotherapeutic drugs has been observed in a variety of tumor types, including common gynecological malignancies such as ovarian cancer [17], cervical cancer [18], and uterine serous carcinoma, a form of type II EC [13]. Consistent with these previous findings, JQ1 reduced cell viability and colony formation in four EC cell lines. JQ1 induces cell apoptosis by promoting an imbalance of mitochondrial apoptotic pathway proteins (Bcl2 and Bax) and enhancing the expression of apoptotic proteins such as cleaved caspase-3 and cleaved PARP. Moreover, BRD4 knockdown suppressed the proliferation of EC cells. These results suggest that the effects of JQ1 may be related to suppression of BRD4 function.

The EC cell lines HEC-1A and Ishikawa are both derived from type I EC [19]. Gene mutations in PIK3CA, PIK3R1, and PTEN are commonly detected in type I EC and may lead to activation of the PI3K/Akt/mTOR pathway [20], which is central to cell growth and proliferation in cancer [21]. In the present study, JQ1 suppressed the expression of PI3K and phosphorylation of AKT and mTOR, supporting the potential application of JQ1 as a potentially novel regimen for the treatment of EC.

In addition to triggering apoptosis, JQ1 suppressed EC cell growth by inducing G1 cell cycle arrest. JQ1 has been reported to cause human neuroblastoma cell cycle arrest in G1 phase mainly by inhibiting the MYCN and mTOR signaling pathways [22]. JQ1 also decreases the proportion of cells in G2 phase and increases the share of cells in G1 phase in a preclinical model of pancreatic cancer [23]. Our investigation of the mechanism of JQ1 showed that treatment with JQ1 alone significantly downregulated the expression levels of CDC25A, CyclinD1, and CDK4, inhibited the activity of Rb, and upregulated the expression of P21, a cell cycle-related factor that mainly suppresses the activity of CDK2/CDK4. CDC25A has dual phosphatase activity and catalyzes the activation of CDK, a cell cycle regulator that plays an important role in the cell cycle transition and mitosis. CDK4 is a member of the serine/threonine kinase family, and the CDK4/CyclinD1 complex regulates the evolution of G1 phase. Our results thus provide insights on the mechanism by which JQ1 regulates the cell cycle in EC.

The four EC cell lines examined in this study varied in their sensitivity to JQ1, and further experiments suggested that this sensitivity may be correlated with expression level of BRD4. As an epigenetic reader, BRD4 activates the transcription of a myriad of genes, including the oncogene c-Myc. Studies have shown that inhibition of c-Myc expression is an essential mechanism by which JQ1 suppresses the progression of various tumors through BRD4 inhibition [24, 25]. Overexpression and amplification of c-Myc is commonly observed in EC [26], and thus c-Myc may be a novel therapeutic target for EC. In our study, dose-dependent and time-dependent decreases in c-Myc expression were observed in cells treated with JQ1. In addition, the level of downregulation of c-Myc using siRNA transfection was approximately equivalent to the level of suppression of cell proliferation. These data imply that the cytotoxic effects of JQ1 in EC cell lines are at least partially mediated by c-Myc.

## Conclusion

In summary, our findings demonstrate that BRD4 is a potential tumor biomarker for EC. The BRD4-specific inhibitor JQ1 is a well-tolerated regimen for treating EC. Furthermore, our data indicate that the antitumor mechanism of JQ1 includes induction of apoptosis and cell cycle arrest in G1 phase through the PI3K/AKT/mTOR pathway and downregulation of c-Myc.

## Supplementary Information

**Additional file 1: Fig. S1**: JQ1 did not influence the cell cycle of HEC-1A cells. (A) HEC-1A cells were treated with DMSO, 2.5µM, 5µM and 10µM JQ1 for 24 hours. Cells were stained with PI and analyzed by flow cytometry. (B) Data are shown in the histogram. Data shown are mean ± SD from three independent experiments

## Data Availability

The data and material used to support the findings of this study are available from the corresponding author upon request.

## Abbreviations

EC: Endometrial cancer
BRD4: Bromodomain 4
BET: Bromodomain and extraterminal
TCGA: The cancer genome atlas
GEO: Gene expression omnibus
OS: Overall survival
IHC: Immunohistochemistry
siRNA: small interfering RNA
p-TEFb: positive transcription elongation factor
DMSO: Dimethyl sulfoxide
MTT: 3-(4,5-Dimethylthiazol-2-yl)-2,5-diphenyltetrazolium bromide
FBS: Fetal bovine serum
SD: Standard deviation
