# Supplementary figures and images for: The BRD4 inhibitor JQ1 suppresses tumor growth by reducing c-Myc expression in endometrial cancer
Source: J Transl Med. 2022 Jul 28;20:336. doi: 10.1186/s12967-022-03545-x (PMC9331486; doi:10.1186/s12967-022-03545-x)

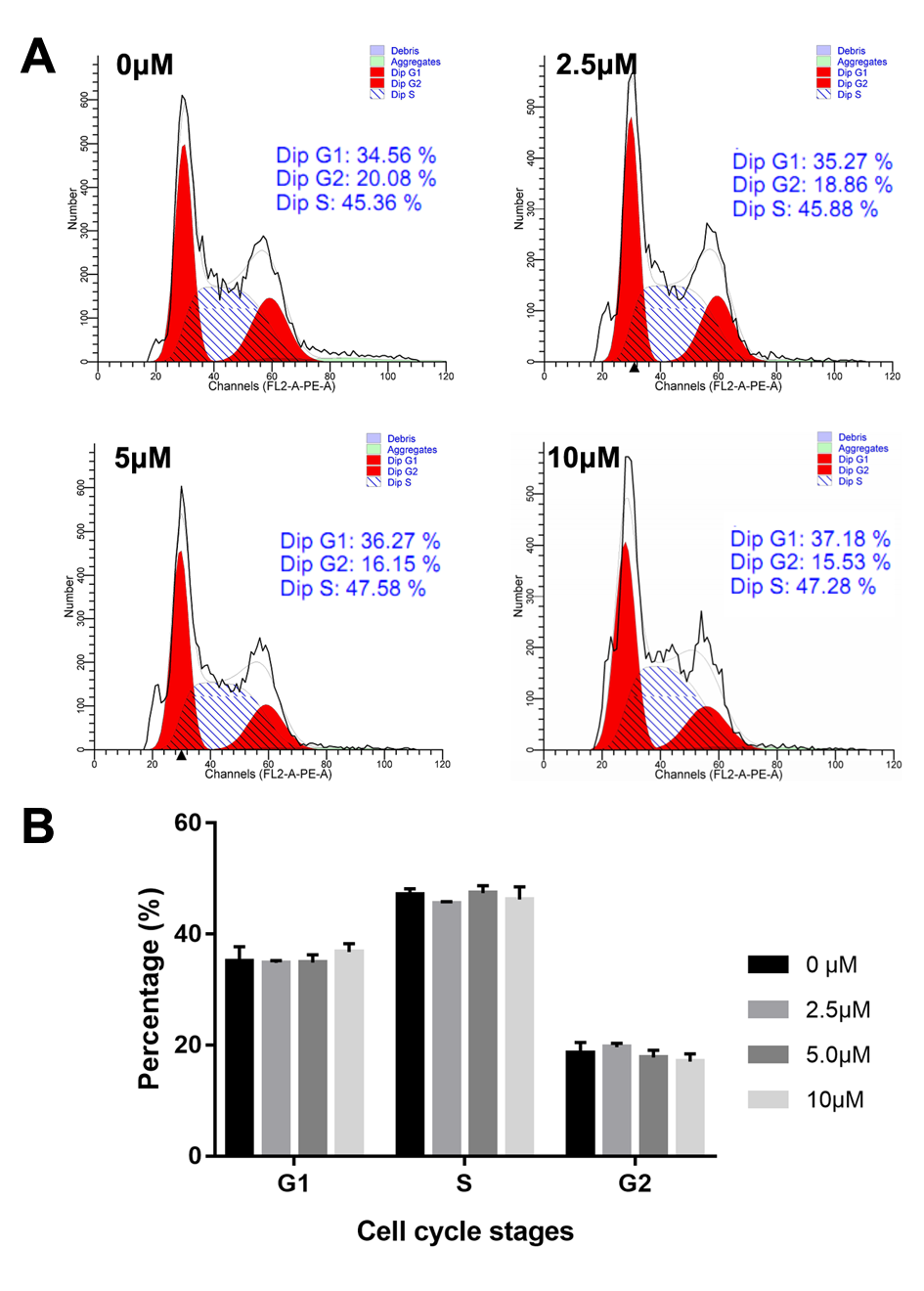

Supplement: Supplementary file 1 — Additional file 1: Fig. S1: JQ1 did not influence the cell cycle of HEC-1A cells. (A) HEC-1A cells were treated with DMSO, 2.5µM, 5µM and 10µM JQ1 for 24 hours. Cells were stained with PI and analyzed by flow cytometry. (B) Data are shown in the histogram. Data shown are mean ± SD from three independent experiments [file 12967_2022_3545_MOESM1_ESM.tif]
